# Reclassifying ovarian cancer: origins, subtypes and resistance to therapy

**DOI:** 10.1186/1897-4287-10-S2-A35

**Published:** 2012-04-12

**Authors:** DDL Bowtell

**Affiliations:** 1 Peter MacCallum Cancer Centre, Research Division, St Andrews Place, East Melbourne, Victoria, Australia

## 

Recent pathological and molecular studies have forced a very significant re-evaluation of the conventional classification of EOC. Microarray and other molecular experiments demonstrate that EOC is a series of molecularly distinct diseases that individually bear more resemblance to certain non-ovarian cancers than they do to each other. Ovarian cancer really represents a spectrum of distinct diseases that share an anatomical location. The presentation focuses on the increasing understanding of the molecular differences between and within different ovarian cancer histotypes. Particular attention is given to high-grade ovarian serous cancers, which account for about two thirds of ovarian cancer deaths, and ovarian clear cell cancers, a tumour type with generally poor response to platinum-based therapy.

Using both gene expression (GE) and DNA copy number (CN) analyses, we have defined novel molecular subtypes of high-grade serous cancers [1]. The molecular subtypes are robustly represented in multiple datasets and are associated with distinct clinical outcomes, and therefore appear to be biologically meaningful. Our efforts to understand the drivers of molecular subtypes of high-grade serous will be discussed [2,3]. A clear cell cohort was analysed using GE and CN analyses, demonstrating deregulation of receptor tyrosine kinases and cytokine pathways [4]. In particular, deregulation of IL6/STAT3/HIF pathway and its targeting in a clinical setting will be described.

Platinum remains the mainstay of treatment for high-grade serous cancers, however, about 20% of patients fail initial treatment and of those that respond, the majority relapse within 2 years and progressively develop resistance to treatment. We have identified mechanisms of primary treatment failure [5,6] and are currently analysing paired primary and relapse samples to determine mechanisms of acquired treatment failure as part of the ICGC project. Studies in treatment resistance will be discussed.
